# Genetic variability, heritability and association among yield components and proximate composition of neglected and underutilized Bambara groundnut [*Vigna subterranea* (L) Verdc] accessions for varietal development in Ghana

**DOI:** 10.1016/j.heliyon.2022.e09691

**Published:** 2022-06-09

**Authors:** Esther Fobi Donkor, Remember Roger Adjei, Braima Amadu, Amanda Sarfo Boateng

**Affiliations:** a Department of Horticulture and Crop Production, School of Agriculture and Technology, Dormaa-Ahenkro Campus, University of Energy and Natural Resources, P.O Box 214, Sunyani, Ghana; b Council for Scientific and Industrial Research-Crops Research Institute (CSRI-CRI), P.O. Box 3785, Fumesua-Kumasi, Ghana

**Keywords:** Bambara groundnut, Food security, Path analysis, Proximate composition, Underutilized crop

## Abstract

Bambara groundnut is an underutilized crop in Ghana with a lot of potential that can be harnessed to contribute to food and nutritional security in Ghana and Sub-Saharan African countries. The recent experiment was conducted to assess the genetic variability, heritability, and association among the yield components and the proximate composition of the Bambara groundnuts. Twenty-five (25) accessions of Bambara groundnut were sourced from different agro-ecological zones. The results from the analysis of variance showed a highly significant difference (p < 0.001) among almost all the traits studied. The estimate of the broad-sense heritability ranged from 12.50% to 84.458% for seed width and economic yield respectively. High heritability values accompanied by high GA were recorded for hundred seed weight (g), economic yield (g), biological yield (g), harvest index, and yield (kg). The separation of correlation coefficients into direct and indirect effects of component traits for yield traits revealed that traits such as economic yield and pod weight which were positively correlated with yield and exerted a high positive direct effect on yield could be selected for varietal development of Bambara groundnut in Ghana. The results from the proximate composition suggest that the Bambara groundnut accessions used in this study have high nutritional potentials that could serve in the alleviation of food security and nutritional problems when these accessions are selected for varietal development.

## 1 Introduction

In Ghana, Bambara groundnut (*Vigna subterranea* (L) Verdc) is a neglected and underutilized crop without any released variety (Adu-Dapaah et al., 2014). Farmers grow Bambara groundnut primarily as a subsistence crop and, to a lesser extent, as a source of revenue. However, because of its drought resistance, the crop has great potential to contribute to food security. This is especially significant in Sub-Saharan Africa and certain parts of the country where rainfall is insufficient to support most leguminous crops (Doku and Karikari 1971). The popularization of Bambara groundnut cultivation and increase in its production could also provide farmers with substantial income (Massawe et al., 2016).

Bambara groundnut is a native African crop that is cultivated around the continent of Africa from Senegal to Kenya and from Sahara to South Africa (Basu et al., 2007). The crop belongs to the *fabaceae* family and sub-family *papilionoidaeae* (Aremu et al., 2006; PROTA 2006). Among the cultivated legumes, Bambara groundnut is one of the most important crops after cowpea and groundnut (Oparaeke and Bunmi 2006). The crop is highly nutritious with an adequate amount of macro and micronutrients (Okonkwo and Opara 2010; Oyeyinka et al., 2018). The fresh beans are boiled with salt and pepper and consumed as a snack in several West African countries. The beans of Bambara groundnut are roasted, crushed, and used to make soup in East Africa (Adu-Dapaah and Sangwan 2004). According to Linnemann (1990), the seeds of Bambara groundnut can be made into a floor which is used in the manufacturing of bread. The milk from Bambara groundnut is mostly consumed more as compared to those prepared from other pulses because of its colour and flavour (Goli 1997). The haulms and the leaves are of important nutritional value when used for animal feed (Rassel 1960; Doku and Karikari 1971).

With the advent of global climate degradation on food security, the Bambara groundnut is a potential crop that can guarantee crop survival under harsh environmental conditions (Muhammad et al., 2020). It contributes to soil nitrogen fixation through the process fixing of atmospheric nitrogen through symbiosis with rhizobium bacteria and it is therefore valuable in intercropping and crop rotations (Mohale et al., 2014). Bambara groundnut is also droughts tolerant of cultivated legumes and is more tolerant to soils that are infertile than other legumes making it a potential food security crop (Berchie et al., 2010).

Despite its nutritional and agronomic benefits, it is a paradox that the Bambara groundnut is a native African crop that produces nearly all the entirely balanced food components, is easy to farm, and requires little soil, should be confined to its continent (Doku, 1996) with no released variants. In Ghana, the Bambara groundnut is still cultivated as landraces without any genetic improvement with its associated low yields (Adu-Dapaah et al., 2014, Alake and Alake, 2016). Researchers have however identified many beneficial traits for improvements of the crop (Basu et al., 2007, Karikari, 2000, Mwale et al., 2007, Ntundu et al., 2006). For effective utilization of available Bambara groundnut germplasm in breeding programs, there is the need to assess the genetic variability among the accessions and also determine the traits which are positively correlated with seed yield to assist breeders to make an informed decision on traits of choice for selection of parental lines (Aliyu et al., 2016, Hoque et al., 2015). Information on the components of variation and character association of various component traits with yield is also of prime importance in breeding programs (Gour et al., 2017, Veni et al., 2013). The partitioning of total variation into phenotypic, genotypic, and environmental components assists to determines the magnitude of the components for the traits and provides vital information on the gene action controlling the inheritance of the traits which is important in deciding the breeding procedure to use in the improvement of the trait. The information on heritability and genetic advance may also assist in the selection of individual traits. Correlation analysis among the traits also determines the relative contribution of the traits, therefore, the path coefficient analysis is done to give an idea of the direct and indirect contribution of a trait towards the yield and this allows the breeder to rank the traits according to their contribution (Dewey and Lu, 1959). The objective of the study was therefore to assess the genetic variability among Bambara groundnut accessions in Ghana based on yield components and to determine the heritability and association among the traits. Also, to determine the proximate composition of the Bambara groundnuts.

## 2 Materials and methods

**Plate 1:**
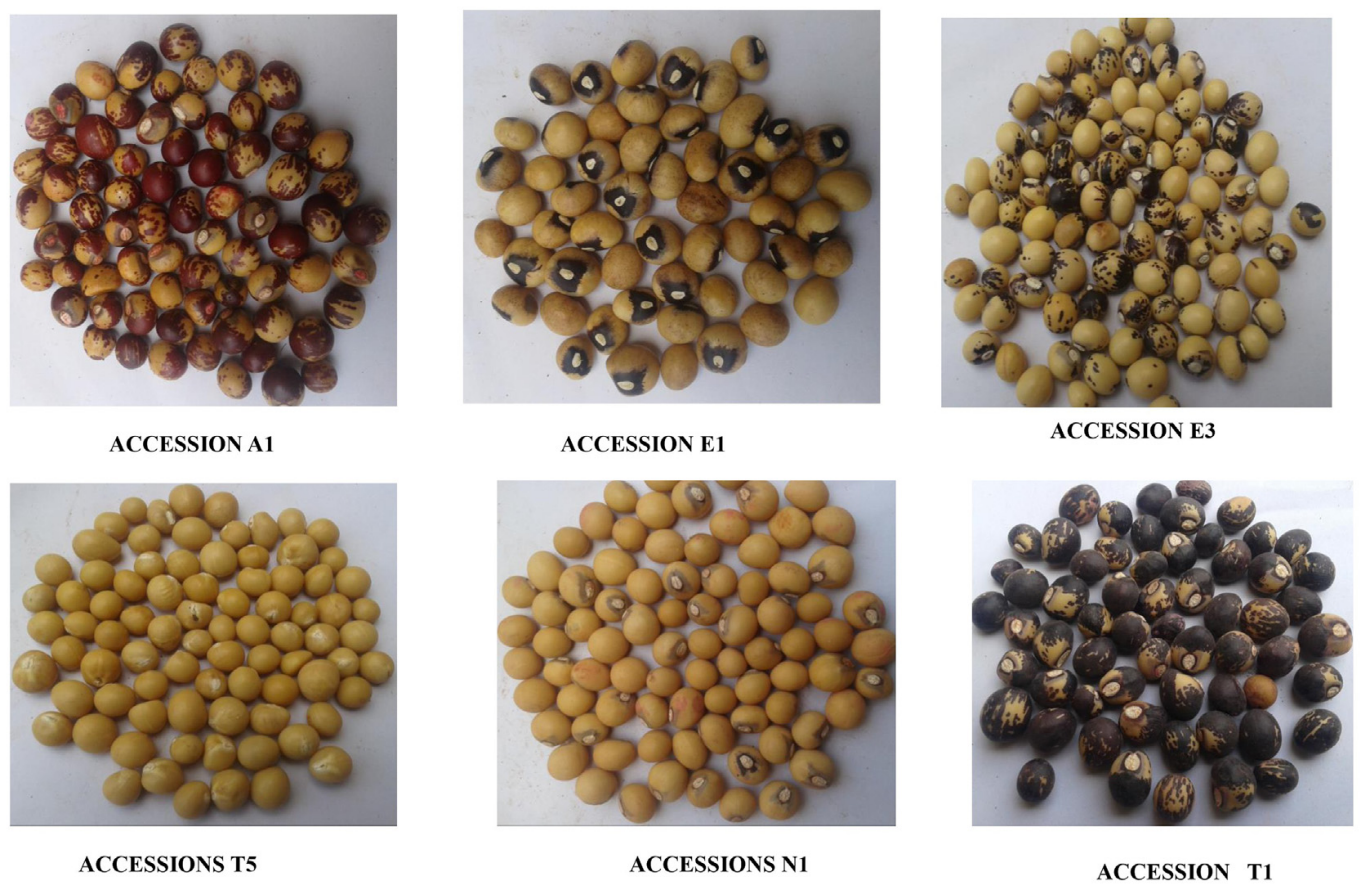
Some of the Bambara groundnut accession.

Twenty-five (25) Bambara groundnut accessions were collected from farmers and traders at key marketplaces in Ghana's primary growing areas, including the Northern, Upper East, Upper West, Ashanti, and Bono East regions. The accession names began with the initial letter of the town from which they were taken, followed by a number to distinguish them if more than one accession was obtained from that town (Table 1, Plate 1). The accessions were planted in a Randomized Complete Block Design with three (3) replications at the multipurpose nursery of the College of Agriculture, Akenten Appiah Minka University of Skills Training and Entrepreneurship Development, Asante Mampong campus (longitude 01^0^241^0^ W, latitude 07^0^04^0^ N, and altitude 457.7 m). Each accession was sown in a plot at a planting distance of 50 cm between rows and 25 cm within rows with 1 seed per hill and 30 plants per plot. All the recommended agronomical practices were carried out to raise a good crop in the season. Data were taken on five (5) randomly picked plants within the middle rows of each plot for yield and yield components. The traits studied were seed length and width [mm]**,** number of immature pods/plants**,** number of pods with two (2) seeds/plant**,** pod length and width (mm), 100 seed weight (g)**,** yield/plot (kg) is the total pod weight divided by the number of plants harvested, economic yield (g), after harvest, the shelled nuts from each of the 5 selected plants from each plot was oven-dried at 80 °C to constant mass and the weight recorded for each plant, biological yield (g) and harvest index (%).

**Table 1:** Description of Bambara groundnut accessions used for the research.

S/N	Name	Source	Region	Description
1	B1	Bawku	Upper East	Dark brown seeds with red mottling and white eyes
2	B2	Bawku	Upper East	Plain Red seeds with white eyes
3	B3	Bawku	Upper East	Creamy seeds with dark black stripes and white eyes surrounded by black colour
4	B4	Bawku	Upper East	Creamy seeds with white eyes surrounded by red colour
5	B5	Bawku	Upper East	Creamy seeds with white eyes surrounded by pale blue colour.
6	B6	Bawku	Upper East	Plain brown seeds with white eyes
7	N1	Navorongo	Upper East	Creamy seeds with white eyes surrounded by pale blue colour.
8	E1	Ejura	Ashanti	Brown seeds with white eyes surrounded by black eyes
9	E2	Ejura	Ashanti	Brown seeds with black mottling and white eyes
10	E3	Ejura	Ashanti	Creamy seeds with black mottling and white eyes with thin black colour surrounding it
11	E4	Ejura	Ashanti	Coffee colored seeds with white eyes
12	A1	Attebubu	Bono East	Creamy seed accompanied with red mottling and white eyes with thin red colour.
13	T1	Tatale	Northern	Creamy seed accompanied with red mottling and white eyes with thin red colour.
14	A3	Attebubu	Bono East	Creamy seeds with white eyes surrounded by black colour.
15	A4	Attebubu	Bono East	Creamy seeds with red mottling and white eyes surrounded by pale blue colour.
16	N2	Navorongo	Upper East	Brown seeds with red mottling and white eyes surrounded by dark red colour.
17	N3	Navorongo	Upper East	Plain Coffee seeds with white eyes
18	W1	Wa	Upper West	Dark red seeds with white eyes
19	N5	Navorongo	Upper East	Creamy seeds with black eyes
20	N6	Navorongo	Upper East	Light coffee seeds with white eyes surrounded by black colour
21	T2	Tatale	Northern	Creamy seeds with white eyes surrounded by black
22	T3	Tatale	Northern	Plain brown seeds with white eyes
23	T4	Tatale	Northern	Light coffee with white eyes surrounded by black colour
24	T5	Tatale	Northern	Completely Cream with white eyes
25	W2	Wa	Upper West	Completely black with white eyes

The shelling percentage was calculated as; $100\;x\frac{weight\;of\;seeds\;\left(g\right)}{weight\;of\;pods\;\left(g\right)}$ for the pods of 5 randomly picked plants for each plot.

### 2.1 Data analysis

All the data collected from the experiment were subjected to Analysis of Variance (ANOVA) using the R statistical software. Separation of the treatment means was done using Tukey's Honest Significant Difference test at 95% confidence level. Based on the formula suggested by Prasad et al. (1981), the phenotypic, genotypic, and environmental variances were estimated as follows:

$$\text{Genotypic ​variance}\left({\text{σ}}^{2}\text{g}\right)=\frac{MSG-MSE}{r}$$

$$\text{Phenotypic ​variance}\left({\text{σ}}^{2}\text{ph}\right)={\text{σ}}^{2}\text{g ​}+{\text{σ}}^{2}e$$

$$\text{Error ​variance}\left({\text{σ}}^{2}\text{e}\right)=\frac{MSE}{r}$$

where:

MSG = Genotypic Mean Squares,
MSE = Error Mean Square and
r = number of replications,
σ^2^e = error variance and
σ^2^g = genotypic variance
σ^2^ph = phenotypic variance

following the formulae suggested by Burton (1952), Johnson et al. (1955), Allard (1961), and Kumar et al. (1985), the various variance component was used in calculating the Genotypic Coefficient of Variability (GCV), Phenotypic Coefficient of Variability (PCV), Broad Sense Heritability (h^2^) and the Genetic Advance (GA) as stated below:

$$\text{Genotypic ​coefficient ​of ​variability ​}\left(\text{GCV ​\%}\right)=GCV=\frac{\sqrt{\sigma^{2}g}}{\bar{X}}x\;100$$

$$\text{Phenotypic ​coefficient ​of ​variability ​}\left(\text{PCV ​\%}\right)=PCV=\frac{\sqrt{\sigma^{2}P}}{\bar{X}}x100$$

Where σ^2^g and σ^2^ph are the genotypic and phenotypic variations respectively and.

X = grand mean for the character under consideration.

$$\text{Heritability ​}\left(\text{broad}-\text{sense}\right)={\text{h}}^{2}=\frac{{\text{σ}}^{2}\text{g}}{{\text{σ}}^{2}\text{ph}}\times 100$$

$$\text{Genetic}\;\text{Advance}=\text{heritability}\times \text{K}\times \sqrt{{\text{σ}}^{2}}\text{ph}$$

K (selection differential expressed in phenotypic standard deviations when 5% selection intensity is used) = 2.06.

The correlation coefficient was calculated for all possible combinations of characters as per the procedure outlined by Miller et al. (1958). Path analysis was also carried out based on the procedure formulated by Dewey and Lu (1959) using INDOSTAT statistical package. The proximate composition of Bambara groundnuts was analyzed using the formulae described by the Association of Official Analytical Chemists (AOAC) (1995).

## 3 Results and discussion

### 3.1 Analysis of variance

The analysis of variance conducted revealed highly significant differences (p < 0.01) among the twenty-five (25) Bambara groundnut accessions for most of the yield components except seed length, pod length, seed width, and pod width (Table 2). This shows the occurrence of considerable genetic variability among the experimental materials, giving room for the selection of superior accessions which could be used as parental lines for varietal development of Bambara groundnut in Ghana. The high level of genetic variability among the accessions gives a greater chance for the selection of desirable traits and could be effective in heterosis breeding (Oladosu et al., 2014; Donkor et al., 2020). Several research has been published on the phenotypic diversity of the Bambara groundnut. For example, the result obtained in our study is similar to the work of Shegro et al. (2013) for the quantitative traits in Bambara groundnuts. Abu and Buah (2011) also reported phenotypic and genotypic diversity among Bambara groundnut accessions in Ghana. Muhammad et al. (2020); Aliyu et al. (2016) and Toure et al. (2012) reported on diversity among Bambara groundnut collections. The accessions showed considerable diversity among the yield and yield component traits.

**Table 2:** Analysis of variance for yield and yield components of Bambara groundnut accessions.

Characters	Replication	Genotype	Error
	df = 2	df = 24	df = 48
100SW(g)	13.545	190.956∗∗∗	50.857
EY(g)	0.253	29.424∗∗∗	4.573
BY(g)	3.293	205.056∗∗∗	58.932
HI	4.745	188.0615∗∗∗	61.565
SH%	142.960	157.677∗	91.094
PW2S	0.973	7.587∗∗∗	1.432
PL (mm)	0.006	0.012	0.017
PW (mm)	0.011	0.014	0.011
SL (mm)	0.005	0.014	0.015
SW (mm)	0.0044	0.008	0.007
IMPODS/PLT	1.44	3.891∗∗	1.718
PODS/PLT	3.88	83.513∗∗∗	25.963
Yield (Kg)	46.72	1353.703∗∗∗	363.887

∗ = P < 0.05, ∗∗ = P < 0.01, ∗∗∗ = P < 0.001, 100SW(g) = hundred seed weight, EY(g) = economic yield, BY(g) = biological yield, HI = harvest index, SH% = shelling percentage, PW2S = pods with two seed, PL (mm) = pod length, PW (mm) = pod width, SL (mm) = seed length, SW (mm) = seed width, IMPODS/PLT = immature pods/plant, PODS/PLT = - total pods/plant, Yield(kg) = yield/plot, df = degree of freedom.

Table 3 shows the overall mean performance of the 25 accessions of Bambara groundnuts. Bawku 5 (B5) recorded the highest mean of 69 g for 100 seed weight and the least was recorded in Navorongo 3 (N3) (36.1 g). The highest economic yield of the Bambara groundnut was observed in the accessions collected from Tatale 4 (T4) (15.3) while the least economic yield was recorded by Tatale 3 (T3) (4.3). The biological yield of the Bambara groundnut accession collected was highest for Navorongo 6 (N6) (63.7) and lowest for accession collected from Navorongo 1 (23.0). Accession B1 recorded the highest harvest index of 47.4 and accession N6 recorded the least harvest index of 15.1. The shelling percentage ranged from 84.3 to 50.8% and accession B3 recorded the highest and accession N1 recorded the least. Accession T2 and B3 recorded the highest number of pods with two seeds (5) and accessions Ejura 2, B2, B1, W1, E4, and N2 recorded the lowest number of pods with two seeds (0). The longest pod length was recorded in accession B3 and B1 (1.6 mm) whereas the lowest was observed in T2 (1.3 mm). With regards to the pod width, accession N1 and N6 recorded the least. The longest seed length was recorded in accession B2 (1.2 mm) and the shortest was observed in 16 (0.9 mm). The seed width was widest for the accessions E3, B1, and B2 (0.9 mm) whereas the smallest seed width of 0.7 mm was recorded in accessions E1, N1, N5, and N6. The number of immature pods per plant was highest for accession E2 (5.3) followed by W1 and N5 (5.0) and the least was counted in accession W2 (0.7). Accession W1 recorded the highest number of total pods per plant (27.0) followed by accession B1 (25.7) and the least total number of pods per plant was recorded in accession N6 (7.0). Accession B3 recorded the highest yield per plot (104.0 kg) while accession N1 recorded the least (16.7 kg).

**Table 3:** Overall mean performance of the yield and yield component traits of Bambara groundnut.

	100 SW(g)	EY(g)	BY(g)	HI	SH %	PW2S	PL (mm)	PW (mm)	SL (mm)	SW (mm)	IMPODS/PLT	PODS/PLT	Yield (kg)
B1	42.5bc	15.0ab	35.3bc	47.4 a	75.9 a	0.0 c	1.6 a	1.1 a	1.0 a	0.9 a	3.3 ab	25.7 ab	69.7 abcd
B2	46.8abc	8.0 cdef	35.3bc	23.6 abc	72.6 a	0.0 c	1.5 a	1.1 a	1.2 a	0.9 a	2.3 ab	18.0 abc	54.0 abcd
B3	50.0abc	6.7 def	38.0bc	17.9 bc	76.9 a	5.0 a	1.6 a	1.0 a	1.0 a	0.8 a	3.0 ab	19.0 abc	104.0 a
B4	40.0c	14.3 abc	38.3bc	40.3 ab	70.7 a	2.0 abc	1.5 a	1.0 a	1.0 a	0.8 a	1.7 ab	8.3 c	45.0 abcd
B5	69.0a	10.0 abcdef	50.0ab	20.2 bc	55.0 a	2.7 abc	1.5 a	1.1 a	1.0 a	0.8 a	4.3 ab	16.3 abc	33.7 bcd
B6	44.6bc	8.3 bcdef	38.3bc	21.6 bc	71.5 a	3.3 abc	1.5 a	1.1 a	1.0 a	0.8 a	3.3 ab	19.7 abc	50.3 abcd
N1	56.4abc	6.0 def	23.0c	25.6 abc	50.8 a	3.3 abc	1.4 a	0.9 a	1.0 a	0.7 a	1.7 ab	18.3 abc	16.7 d
E1	39.3c	7.7 cdef	41.0 abc	19.2 bc	65.3 a	2.7 abc	1.4 a	1.1 a	1.1 a	0.7 a	4.3 ab	14.0 abc	50.3 abcd
E2	58.4abc	5.3 f	30.0 bc	17.7 bc	65.1 a	0.0 c	1.5 a	1.1 a	1.1 a	0.8 a	5.3 a	14.7 abc	47.0 abcd
E3	55.0abc	6.0 def	34.0 bc	17.9 bc	66.1 a	2.3 abc	1.5 a	1.1 a	1.2 a	0.9 a	3.7 ab	18.0 abc	37.7 bcd
E4	46.4abc	7.0 def	36.7 bc	19.8 bc	68.2 a	0.0 c	1.4 a	1.1 a	1.0 a	0.8 a	3.7 ab	18.7 abc	33.3 cd
A1	39.5c	12.7 abcd	35.3 bc	36.1 abc	68.6 a	1.0 bc	1.5 a	1.1 a	1.0 a	0.8 a	4.0 ab	21.7 abc	38.7 bcd
T1	47.7abc	10.0 abcdef	37.7 bc	26.8 abc	73.1 a	2.0 abc	1.5 a	1.0 a	1.0 a	0.8 a	4.0 ab	13.7 abc	54.3 abcd
A3	39.3c	6.7 def	33.3 bc	20.5 bc	75.8 a	3.0 abc	1.4 a	1.1 a	1.1 a	0.8 a	2.3 ab	7.0 c	54.7 abcd
A4	44.7bc	9.3 abcdef	41.7 abc	22.6abc	72.3 a	2.7 abc	1.4 a	1.0 a	1.0 a	0.8 a	4.3 ab	17.7 abc	41.3 bcd
N2	36.1c	8.0 cdef	36.7 bc	21.6bc	62.7 a	0.0 c	1.5 a	1.1 a	0.9 a	0.8 a	4.0 ab	20.7 abc	52.0 abcd
N3	46.0bc	5.3 f	33.7 bc	16.1bc	70.9 a	4.0 ab	1.4 a	1.1 a	1.1 a	0.8 a	3.3 ab	20.3 abc	26.7 cd
W1	42.1bc	6.3 def	36.7 bc	17.4bc	75.3 a	0.0 c	1.5 a	1.0 a	1.1 a	0.8 a	5.0 a	27.0 a	36.7 bcd
N5	44.7bc	7.0 def	34.7 bc	20.5bc	72.5 a	2.0 abc	1.5 a	1.0 a	1.0 a	0.7 a	5.0 a	21.0 abc	75.7 abcd
N6	45.5bc	9.0 abcdef	63.7 a	15.1c	62.9 a	1.0 bc	1.4 a	0.9 a	1.0 a	0.7 a	3.0 ab	7.0 c	58.7 abcd
T2	41.5bc	5.7 ef	29.7 bc	20.2bc	63.4 a	5.0 a	1.3 a	1.0 a	1.0 a	0.8 a	4.0 ab	17.7 abc	64.3 abcd
T3	42.2bc	4.3 f	23.0 c	18.7bc	62.7 a	3.7 abc	1.5 a	1.0 a	1.1 a	0.8 a	4.7 ab	17.3 abc	94.0 ab
T4	44.0bc	15.3 a	45.7 abc	33.7abc	84.3 a	3.7 abc	1.4 a	1.1 a	1.0 a	0.8 a	2.7 ab	16.3 abc	80.7 abc
T5	63.1ab	8.3 bcdef	39.7 abc	21.2 bc	61.5 a	1.7 abc	1.4 a	1.1 a	1.1 a	0.8 a	3.3 ab	10.0 bc	76.0 abcd
W2	52.1abc	12.3 abcde	47.0 abc	26.3 abc	65.8 a	3.3 abc	1.5 a	1.1 a	1.1 a	0.8 a	0.7 b	9.0 c	78.7 abc
**Mean**	**47.09**	**8.59**	**37.53**	**23.52**	**78**	**2.18**	**12.2**	**1.02**	**1.05**	**0.79**	**4.00**	**16.68**	**55.0**
**CV**	**15.1**	**24.9**	**20.5**	**23.4**	**NS**	**25.1**	**NS**	**NS**	**NS**	**NS**	**27.7**	**30.5**	**30.7**

Means that do not share a letter are significantly different. 100SW(g) = hundred seed weight, EY(g)= economic yield, BY(g)= biological yield, HI= harvest index, SH%= shelling percentage, PW2S= pods with two seed, PL (mm)= pod length, PW (mm)= pod width, SL (mm) = seed length, SW (mm) = seed width, IMPODS/PLT = immature pods/plant, PODS/PLT =- total pods/plant, Yield(kg) = yield/plot.

### 3.2 Estimation of variability

The genotypic variance (GV), error variance (EV), phenotypic variance (PV), genotypic and phenotypic coefficient of variation (GCV and PCV respectively), heritability, and genetic advance for the yield and yield components are presented in Table 4. The PCV and GCV values with less than 10% were categorized as low, 10–20% as moderate, and greater than 20% as high (Sivasubramanian and Menon 1973). The GV was lower than the PV for all the traits while the GV was also greater than the EV for all the studied traits. This indicates the minimal influence of the environment in the expression of the traits. The studies also revealed a higher magnitude of PCV than GCV for all the traits confirming the minimal influence of the environment on the expression of the traits. High to moderate GCV and PCV were recorded for biological yield(g), harvest index, economic yield(g), number of pods with 2 seeds, immature pods/plant, number of pods/plants, and yield (kg). This indicates that applying phenotypic selection to such traits may be effective for the varietal development of high-yielding Bambara groundnuts. The low GCV and PCV recorded for the remaining traits indicates the high influence of the environment in the expression of those traits. Hampannavar et al. (2018); Bhakal and Lal (2017) reported similar results for yield components in groundnut. The heritability (broad sense) estimates also ranged from 12.50% to 84.46% for seed width and economic yield respectively. Genetic advance (GA) also ranged from 0.01 to 31.99 for seed width (mm) and yield (kg) respectively. The highest GA as a percentage of the mean (121.91) was recorded for pods with 2 seeds while the least (1.13) was recorded for the seed length. Per the work of Bello et al. (2012), heritability estimates are useful for breeding quantitative traits because they provide information on the degree to which a particular trait can be inherited by subsequent generations. Heritability values were categorized as low (0–30%), moderate (30–60%) and high (>60%) (Robinson et al., 1949). The combination of heritability estimates and genetic advances would be more useful in predicting genotypes for selection purposes than the use of only the estimate of heritability (Johnson et al., 1955). Also, Panse (1957) reported that the traits that have high heritability and high genetic advance are controlled by addictive genes and therefore will benefit from the selection. High heritability coupled with low genetic advance is a result of the non-addictive gene effects. Our results showed high heritability values accompanied by high GA for hundred seed weight(g), economic yield(g), biological yield(g), harvest index, and yield (kg). This suggests the influence of additive gene action in the control of the traits and therefore phenotypic selection can be used to improve the traits. Kushwah et al. (2016) and Mohan et al. (2013) also reported similar results for yield and its components in groundnut. Low heritability along with low genetic advance was recorded for the pod length (mm), seed length (mm) and seed width (mm) suggesting the influence of non-additive gene action in the expression of the traits therefore phenotypic selection may not be effective in the improvement of these traits. Bhakal and Lal (2017) and John et al. (2012) also reported similar results for groundnut.

**Table 4:** Genetic estimates for yield and yield components in Bambara groundnut.

Characters	σ^2^g	σ^2^ph	σ^2^e	GCV	PCV	H^2^	GA	% GA mean
100SW	46.70	63.65	16.95	14.51	16.94	73.37	12.06	25.61
EY	8.284	9.808	1.524	33.506	36.46	84.458	5.449	63.43
BY	48.7	68.35	19.64	18.59	22.03	71.26	12.14	32.34
HI	42.16	62.68	20.52	27.61	33.66	67.27	10.97	46.64
SH%	22.194	52.559	30.365	6.040	9.29	42.227	6.31	8.09
PW2S	2.05	2.53	0.477	66.01	73.29	81.13	2.66	121.91
PL (mm)	-0.02	0.004	0.006	2.91	4.25	46.9	-0.06	-4.10
PW (mm)	0.001	0.005	0.004	3.10	6.70	21.43	0.03	2.96
SL (mm)	0.042	0.053	0.048	1.95	6.89	79.25	0.01	1.13
SW (mm)	0.001	0.003	0.002	2.311	6.54	12.50	0.013	1.68
IMPODS/PLT	0.72	1.29	0.573	24.46	32.73	55.85	1.31	32.75
PODS/PLT	19.18	27.84	8.653	26.26	31.63	68.92	7.49	19.45
Yield (kg)	329.93	451.23	121.3	33.03	38.62	73.12	31.99	58.17

100SW(g) = hundred seed weight; EY(g) = economic yield; BY(g) = biological yield; HI = harvest index, SH% = shelling percentage, PW2S = pods with two seed; PL(mm) = pod length; PW(mm) = pod width; SL(mm) = seed length; SW(mm) = seed width; IMPODS/PLT = immature pods/plant; PODS/PLT = - total pods/plant; Yield(kg) = yield/plot; σ^2^e = error variance; σ^2^g = genotypic variance; GCV = genotypic coefficient of variability; PCV = phenotypic coefficient of variability; H^2^ = broad sense heritability; GA = genetic advance.

### 3.3 Association among the yield and yield components

The correlation analysis of the yield and yield component for the 25 Bambara groundnut is shown in Table 5. Our results showed a significant and positive relationship between some of the traits of the yield component and the grain yield. For example, the Pearson correlation analysis revealed strong and positive significant association between yield and economic yield (r = 0.201, p < 0.01), harvest index (r = 0.132, p < 0.01), shelling percentage (r = 0.177, p < 0.01), pods with two seeds (r = 0.237, p < 0.05) and number of pods per plant (r = 0.223, p < 0.01) (Table 5). The shelling percentage was positively and significantly correlated with hundred seed weight. The results demonstrated a significant positive relationship between Economic yield and harvest index (r = 0.795, p < 0.001). For improvement in the yield of Bambara groundnut, concentration should be on these traits as improvement in them will increase the yield of Bambara groundnut. Biological yield was also significantly and positively correlated with the economic yield (r = 0.339, p < 0.01) but revealed a negative significant association with Harvest index (r = -0.237, p < 0.01), shelling percentage (r = -0.039, p < 0.01) and pods with 2 seeds (r = -0.052, p < 0.01). Seed width revealed positive and significant correlation with pod length (r = 0.270, p < 0.05), pod width (r = 0.333, p < 0.01) and seed length (r = 0.458, p < 0.001). Khan et al. (2020) and Gao et al. (2020) also recorded a significant positive correlation and contributions between yield and 100 seed weight in Bambara groundnut. Similar results have also been reported by Gbaguidi et al. (2018), Pranesh et al. (2017), and Smita et al. (2014) in Bambara groundnut.

**Table 5:** Correlation among the yield and yield components of Bambara groundnut.

Traits	100SW(g)	EY (g)	BY (g)	HI	SH%	PW2S	PL (mm)	PW (mm)	SL (mm)	SW (mm)	IMPODS/PLT	PODS/PLT	Yield (kg)
100SW(g)	-	0.002∗∗	-0.158	-0.106	-0.337∗∗	0.045	0.132	0.084	0.026	0.106	0.051	-0.211∗	0.006
EY(g)		-	0.339∗∗	0.795∗∗∗	0.134	0.158	0.180	0.109	-0.177	0.031	-0.271∗	-0.096	0.201∗∗
BY(g)			-	-0.237∗	-0.039∗∗	-0.052∗	0.166	-0.106	-0.043	-0.132	-0.174	-0.319	0.080
HI				-	0.154	-0.164	0.125	0.137	-0.179	0.099	-0.156	0.077	0.132∗∗
SH%					-	0.077	0.202	0.154	-0.085	0.021	0.012	0.209	0.177∗∗
PW2S						-	-0.091	-0.035	-0.079	-0.227∗	-0.152	-0.128	0.237∗
PL (mm)							-	0.219	-0.034	0.270∗	-0.119	0.100	0.156
PW (mm)								-	0.143	0.337∗∗	-0.037	-0.009	0.113
SL (mm)									-	0.458∗∗∗	-0.035	-0.147	0.059
SW (mm)										-	0.072	0.032	0.012
IMPODS/PLT											-	0.313∗∗	-0.071
PODS/PLT												-	0.223∗∗
Yield(kg)													-

∗ = P < 0.05, ∗∗ = P < 0.01, ∗∗∗ = P < 0.001, 100SW(g) = hundred seed weight, EY(g) = economic yield, BY(g) = biological yield, HI = harvest index, SH% = shelling percentage, PW2S = pods with two seed, PL(mm) = pod length, PW(mm) = pod width, SL(mm) = seed length, SW(mm) = seed width, IMPODS/PLT = immature pods/plant, PODS/PLT = - total pods/plant, Yield(kg) = yield/plot.

### 3.4 Path analysis

**Figure 1:**
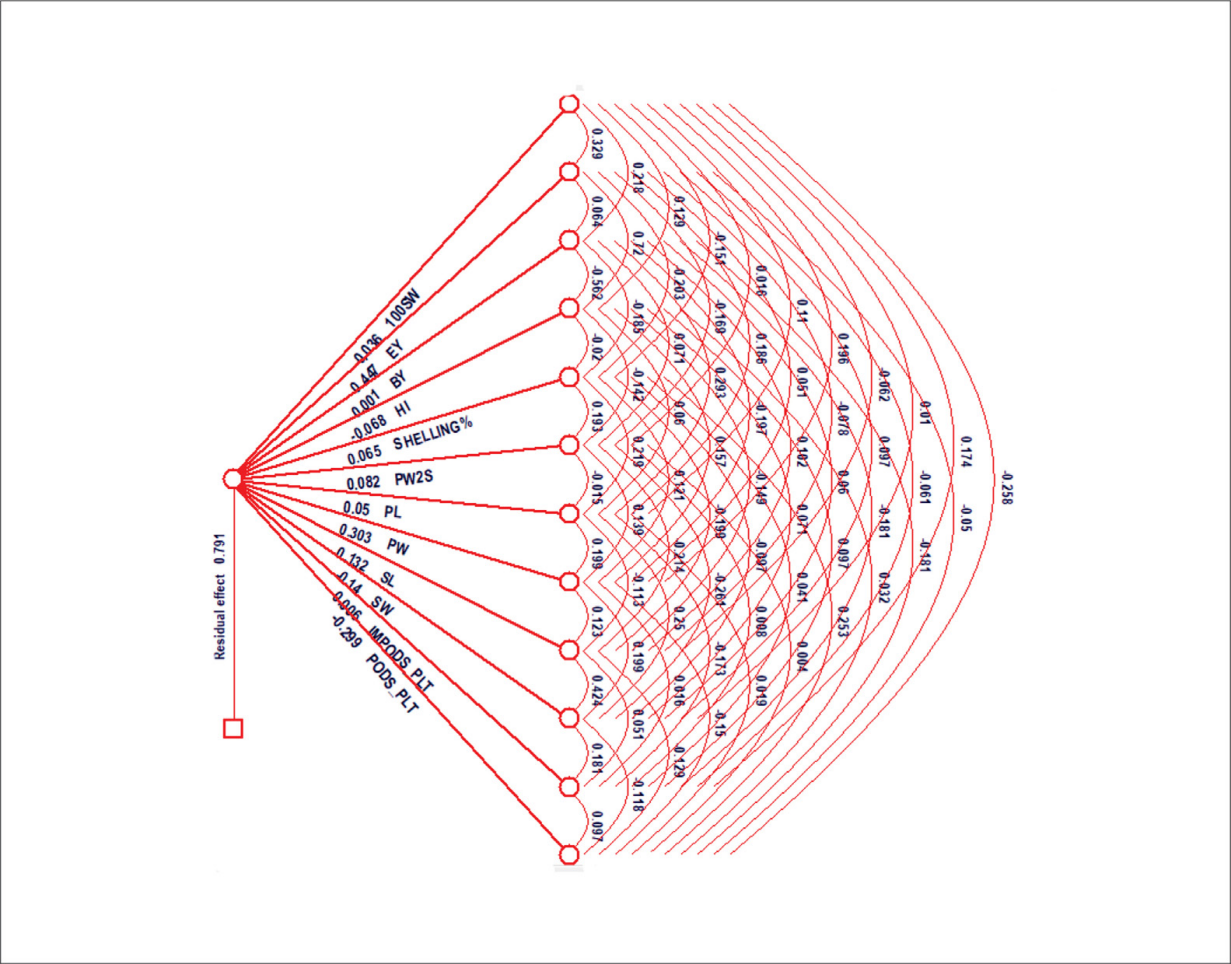
Direct (diagonal) and indirect effects of yield components on yield of Bambara groundnut. 100SW(g) = hundred seed weight, EY(g) = economic yield, BY(g) = biological yield, HI = harvest index, SH% = shelling percentage, PW2S = pods with two seed, PL (mm) = pod length, PW(mm) = pod width, SL (mm) = seed length, SW(mm) = seed width, IMPODS/PLT = immature pods/plant, PODS/PLT = - total pods/plant.

The degree and direction of the association between yield and yield components are significant for determining the important traits that may be adopted in the breeding program as a crop enhancement technique using selective breeding. The use of a correlation matrix alone is not an accurate method of capturing the characteristics of contributing traits to the yield. Path coefficient analysis is one of the most commonly used methods in determining the interaction between traits against yield (Khan et al., 2022). The correlation coefficients of these contributing traits with yield are categorized as direct and indirect effects. The correlation coefficients were divided into direct and indirect effects according to Dewey and Lu (1959) (Figure 1). Lenka and Misra (1973) grouped the path coefficients as negligible (0.00–0.09), low (0.1–0.19), moderate (0.2–0.29) and high (0.3–0.99). The direct and indirect effects of the traits on yield are presented in Figure 1. The direct effects ranged from -0.035 for hundred seed weight to 0.303 for pod weight. with a residual effect of 0.79. Economic yield and pod weight recorded a high direct effect on yield, Seed length recorded a low direct effect on yield. Biological yield, shelling percentage, pods with 2 seeds, pod length, and immature pods per plant recorded negligible positive direct effect. Hundred seed weight, harvesting index, seed width, and pods per plant recorded a negative direct effect with pods per plant having a moderate effect and hundred seed weight negligible effect. Nevertheless, these traits with positive direct effects are considered the key contributors to overall yield. Most of the indirect effects both negative and positive were negligible, however, the harvest index gave the highest positive indirect effect on yield through ecological yield while the highest negative indirect effect was recorded by ecological yield through the harvest index. These characteristics, however, can be used to develop an ideally effective selection index for improving the yield of Bambara groundnut. Traits such as economic yield and harvest index and pods with 2 seeds that were positively correlated with yield and exerted high to a moderate positive direct effect on yield could be selected for varietal development of Bambara groundnut in Ghana. Our results corroborate with the work of Pranesh et al. (2017); Oyiga and Uguru (2011); Misangu et al. (2007) on Bambara groundnut. Mubai et al. (2020) also reported similar results for groundnut accessions.

### 3.5 Proximate composition of Bambara groundnut

The proximate composition of the twenty-five accessions of the Bambara groundnut is presented in Table 6. The parameters determined were crude protein, crude fiber, ether extract, ash, moisture content, nitrogen-free extract, and the energy level The results indicated highly significant differences (p < 0.001) for the proximate composition. Crude protein content ranges from 19.70 to 30.10%, Crude fiber, 2.27–5.81 %, ether extract 6.00–11.00%, ash 2.00–3.50 %, moisture content ranges from 11.00 to 26.00%, nitrogen-free extract ranges from 53.59 to 67.23 %, and the energy level ranges from 36.04 to 37.65 %. The proximate composition of Bambara groundnuts was earlier reported by several authors (Anhwange and Atoo, 2015; Alhassan et al., 2015; Adegboyega et al., 2021). The results from the proximate analysis indicate that the Bambara groundnut is a good source of crude protein, fiber, ether extract, ash, moisture content, and nitrogen-free extracts. However, similar results were obtained for the crude fiber in the study of Alhassan et al. (2015); Anhwange and Atoo (2015). Higher crude fiber is beneficial in improving the general well-being of humans and animals. In addition, crude fiber is an important component in the diet of humans due to its ability to treat constipation, reduce the risk of bowel diseases, lower cholesterol, and also improve the general well-being and health of humans (Anhwange and Atoo, 2015). Further, it is reported that growth retardment, abnormal swelling of the stomach, and muscle wasting is as a result of protein deficiency. The values obtained for the proximate analysis show that the Bambara groundnut is a good source of protein and could be used as a supplement for animal protein.

**Table 6:** Proximate composition of Bambara groundnut (on dry matter basis).

Accession	CP	CF	EE	ASH	MC	NFE	EL
B1	24.10cb	4.35ed	8.00bc	2.50bc	13.00ij	61.05edhgf	36.60ebdacf
B2	30.10a	5.81a	8.00bc	2.50bc	17.50fg	53.59i	36.0927edgf
B3	21.00cd	4.67cbd	11.00a	3.50a	18.50efg	59.83hg	37.64a
B4	24.10cb	4.82b	6.00c	3.00ba	22.500c	62.08edhgcf	35.2630g
B5	24.10cb	4.60cebd	9.00ba	2.00c	17.33fg	60.30ehgf	37.19bdac
B6	21.90cbd	4.44ced	9.00ba	2.50bc	14.50ih	62.16edhgcf	37.07ebdac
N1	24.10cb	4.35ed	8.00bc	2.00c	11.00j	61.55edhgcf	36.78ebdac
E1	21.87cbd	4.65cbd	9.00ba	2.50bc	17.50fg	61.98edhgcf	36.99ebdac
E2	21.87cbd	3.41gfh	7.33bc	3.50a	18.00efg	63.89ebdacf	36.24ebdgcf
E3	24.50b	3.41gfh	9.00ba	3.00ba	7.00k	60.090hgf	37.26bac
E4	22.80cbd	3.41gfh	8.00bc	3.00ba	19.50ef	62.79ebdgcf	36.75ebdac
A1	21.90cbd	3.61gf	8.00bc	3.00ba	22.00dc	3.49ebdagcf	36.67ebdac
T1	22.27cbd	3.53gfh	7.00bc	3.500a	20.00ed	63.70ebdagcf	36.04egf
A3	19.70d	3.33gh	9.00ba	3.00ba	17.50fg	64.97bac	37.28ba
A4	20.100d	3.53gfh	7.00bc	3.00ba	23.00bc	66.37ba	36.22ebdgcf
N2	21.90cbd	3.57gf	7.00bc	3.00ba	25.00ba	4.53bdac	36.20ebdgcf
N3	24.10cb	3.37gfh	7.33bc	2.50bc	17.500fg	62.69ebdhgcf	36.61ebdacf
W1	19.70d	3.57gf	6.00c	3.50a	26.00a	67.23a	35.53gf
N5	24.10cb	3.23h	8.00bc	3.50a	11.67j	61.17edhgcf	36.64ebdacf
N6	21.90cbd	2.22i	8.00bc	3.00ba	12.50ij	64.88bdac	37.17bdac
T2	24.13cb	3.57gf	7.00bc	3.00ba	24.00bac	62.29dhgfe	36.20ebdgcf
T3	22.30cbd	3.70f	7.00bc	3.00ba	24.00bac	64.00ebdac	36.16edgcf
T4	24.90b	4.76cb	9.00ba	2.50bc	16.833g	58.840h	36.95ebdac
T5	24.10cb	4.30e	8.00bc	2.50bc	16.50hg	61.10edhgcf	36.62ebdacf
W2	22.80cbd	2.27i	9.00ba	3.00ba	17.000g	62.93ebdgcf	37.65a
**Grand Mean**	**22.97**	**3.86**	**7.99**	**2.88**	**17.99**	**62.3**	**36.63**
**CV%**	**9.096**	**5.31**	**16.68**	**12.55**	**7.47**	**3.79**	**1.87**

CP = Crude Protein, CF = Crude Fiber, EE = Ether Extract, ASH = Ash, MC = Moisture Content, NFE = Nitrogen Free Extract, EL = Energy Level. Means in the same column followed by the same letter are not significantly different (*P < 0.05*).

### 3.6 Association among the proximate composition

Table 7 shows the character associations among the proximate composition studied for the 25 Bambara groundnut accessions. The Ash content showed positive significant correlation with the moisture content (r = 0.244, p < 0.05) and the Nitrogen Free Extract (r = 0.244, p < 0.05). A positive and significant association was also observed between the Crude fibre and Crude protein (r = 0.358, p < 0.05). The moisture content revealed positive and significant correlation with nitrogen-free extract (r = 0.328, p < 0.05) but was negatively correlated with Energy Level (r = -0.398, p < 0.05). Ether extract showed positive and significant correlation with Energy Level (r = 0.938, p < 0.05) and negative and significant correlation with moisture content (r = -0.351, p < 0.05) and nitrogen-free extract (r = -0.437, p < 0.05). The crude protein revealed negative and significant correlation with Ash Content (r = -0.296, p < 0.05), Moisture Content (r = -0.253, p < 0.05) and nitrogen-free extract (r = -0.844, p < 0.05). Crude fibre also revealed negative and significant correlation with Nitrogen Free Extract (r = - 0.539, p < 0.05) and Ash Content (r = -0.346, p < 0.05). The positive and significant correlations among the traits show that an increase in one particular trait would automatically increase the other trait. For example, the strong significant, and positive correlation between the crude protein and crude fibre; energy level and Ether extract; Ash and moisture content and NFE; and moisture content and NFE of the biochemical traits suggest that these traits can be improved simultaneously. Further, the strong significant and negative correlation between crude protein and Ash, moisture content and NFE; moisture content and energy level; NFE and energy level; crude fibre and NFE and Ash suggests that selection based on the crude fibre content of Bambara groundnut will depress the NFE and ash content of the accession and thus these traits will have to be improved separately. Our findings are in line with Subhash et al. (2008), who reported significant (p > 0.05), positive and negative correlations among some nutrient traits in maize.

**Table 7:** Association among the proximate composition of the Bambara groundnut.

	CP	CF	EE	ASH	MC	NFE	EL
CP	-	0.358∗∗	-0.048	-0.296∗∗	-0.253∗	-0.844∗∗	0.102
CF		-	0.109	-0.346∗∗	0.027	-0.539∗∗	-0.165
EE			-	-0.122	-0.351∗∗	-0.437∗∗	0.938∗∗
ASH				-	0.244∗	0.224∗	-0.226∗
MC					-	0.328∗∗	-0.398∗∗
NFE						-	-0.278∗∗

CP = Crude Protein, CF = Crude Fiber, EE = Ether Extract, ASH = Ash, MC = Moisture Content, NFE = Nitrogen Free Extract, EL = Energy Level.

## 4 Conclusion

Our present study evaluated the genetic variability, heritability, and association among yield components and proximate composition of neglected and underutilized 25 Bambara groundnut [*Vigna subterranea* (L) Verdc] accessions for varietal development in Ghana in terms of 100SW (g), EY (g), BY (g), HI, Shelling%, PW2S, immature pods per plants, pods per plant and yield (kg). Our results revealed significant genetic variability among the Bambara groundnut accessions used for the study. These, however, suggest that there is highly prospective for the selection and screening of the accessions for varietal development to promote food nutrition and security. Accessions W2, T5, T4, T3, N5, and B3 exhibited high yield traits. High heritability accompanied by high GA for the yield components provides room for the use of phenotypic selection for the development of high-yielding varieties of Bambara grounds in Ghana. Also, the positive correlation and high positive direct effects exerted by economic yield and harvest indicate that the yield of Bambara groundnut can be improved by improving these traits. The proximate composition of the accessions showed higher content of crude fiber, protein, ether extract, ash, moisture content, and nitrogen-free extracts which indicates that the Bambara groundnut accessions have the potential to improve nutritional deficiencies and food security if the accessions are properly harnessed for human consumption.

## Declarations

### Author contribution statement

Esther Fobi Donkor: Conceived and designed the experiments; Performed the experiments; Analyzed and interpreted the data; Contributed reagents, materials, analysis tools or data; Wrote the paper.

Remember Roger Adjei; Braima Amadu; Amanda Sarfo Boateng: Conceived and designed the experiments; Analyzed and interpreted the data; Contributed reagents, materials, analysis tools or data; Wrote the paper.

### Funding statement

This research did not receive any specific grant from funding agencies in the public, commercial, or not-for-profit sectors.

### Data availability statement

Data will be made available on request.

### Declaration of interests statement

The authors declare no conflict of interest.

### Additional information

No additional information is available for this paper.
